# Painfully Sensitive: How Sensory Processing Sensitivity Affects Healthy Adolescents’ Perception of Pain

**DOI:** 10.2147/JPR.S473575

**Published:** 2025-02-15

**Authors:** Jana Hochreuter, Susanne Wehrli, Cosima Locher, Francesca Lionetti, Joe Kossowsky, Michael Pluess, Helen Koechlin

**Affiliations:** 1 Department of Psychosomatics and Psychiatry, University Children’s Hospital, University of Zurich, Zurich, Switzerland; 2 Division of Child and Adolescent Health Psychology, Department of Psychology, University of Zurich, Zurich, Switzerland; 3 Children’s Research Centre, University Children’s Hospital Zurich, University of Zurich, Zurich, Switzerland; 4 University Research Priority Program “ITINERARE –Innovative Therapies in Rare Diseases”, University of Zurich, Zurich, Switzerland; 5 Department of Consultation-Liaison Psychiatry and Psychosomatic Medicine, University Hospital Zurich, Zurich, Switzerland; 6 Faculty of Health, University of Plymouth, Plymouth, UK; 7 Department of Neuroscience, Imaging and Clinical Sciences, G. d’Annunzio University of Chieti-Pescara, Chieti, Italy; 8 Department of Anesthesiology, Critical Care and Pain Medicine, Boston Children’s Hospital, Boston, MA, USA; 9 Harvard Medical School, Boston, MA, USA; 10 School of Psychology, University of Surrey, Guildford, UK; 11 Department of Biological and Experimental Psychology, Queen Mary University, London, UK

**Keywords:** experimental pain, sensory processing sensitivity, adolescents

## Abstract

**Objective:**

Sensory processing sensitivity (SPS) describes a common trait characterized by lower sensory threshold, depth of processing, and ease of overstimulation. Low sensory threshold is also potentially important in the context of pain. To date, the relationship between SPS and pain perception has not been investigated, particularly in adolescents. This randomized experimental study aimed to explore whether SPS was associated with pain threshold and tolerance in healthy adolescents. Further, we examined differences in pain perception following positive, negative, or neutral mood induction.

**Methods:**

A total of 100 healthy adolescents aged 16 to 19 years were recruited through schools and online advertisement. Participants completed psychosocial questionnaires and underwent a randomized mood induction (positive, negative, or neutral). Pain levels (in °C) and self-reported pain tolerance and threshold were assessed by an experimental heat pain paradigm. Regression models were applied to determine effects of mood induction on pain perception, while multiple analyses of variance (ANOVAs) were computed to compare sensitivity across groups.

**Results:**

The mood induction significantly influenced self-reported pain ratings, demonstrating a positive interaction coefficient for the positive mood condition (*b* = 1.140, *p* < 0.01). ANOVAs revealed significant differences between sensitivity groups for baseline scores of pain threshold (F_(2,97)_ = 5.009, p < 0.01) and tolerance (F_(2,97)_ = 7.431, p < 0.001).

**Conclusion:**

Our findings reveal that highly sensitive adolescents had the lowest ratings of measured pain tolerance and threshold measures at baseline, suggesting heightened sensitivity to pain. This highlights the importance of considering sensory processing sensitivity in pain research, particularly in adolescent populations.

## Introduction

Pain is a complex, unpleasant sensory and emotional experience that is influenced – to varying degrees – by psychological, social, and biological factors.^1^,^2^ When pain becomes chronic – lasting or recurring for three or more months – it affects up to one in four children and adolescents,^3^,^4^ and is linked to considerable psychological, physical, and social concerns for both the affected individuals and their families.^5^,^6^ Pain, especially chronic pain, in childhood and adolescence tends to persist into adulthood and can predict psychological diseases.^7–10^ Therefore, a deeper understanding of pain mechanisms in adolescence is essential.

Differences in pain processing might be associated with an individual’s general sensitivity, with some individuals being more sensitive to their environmental context than others.^11^ One theoretical framework for the mechanism of variability in Environmental Sensitivity is Sensory Processing Sensitivity (SPS).^12–14^ SPS is a stable personality trait,^13^,^15^ with highly sensitive individuals exhibiting deeper cognitive processing of external stimuli, greater emotional reactivity, and heightened awareness of sensory stimulation.^13^,^16^ Based on recent estimations, around 20%–35% of healthy children and adolescents fall into a high-sensitivity group.^15^ However, in a sample of adolescents who reported chronic pain, the proportion of highly sensitive individuals was much larger with 45.7%, and those in the high-sensitivity group reported higher pain-related distress compared to the medium and low-sensitivity groups.^17^ Characteristics of highly sensitive individuals, specifically low sensory threshold (ie, sensitivity to external stimuli), indicate a potential link to pain vulnerability and altered pain processing.^18^,^19^

Previous studies have found associations between SPS and several other variables that may contribute to pain: Given that highly sensitive individuals experience increased emotional reactivity, ie, a lower threshold required for emotional reactions, leading to more frequent and more intense emotions, the *regulation* of emotions might become more challenging.^13^,^20^,^21^ Maladaptive or dysfunctional emotion regulation, such as suppression or rumination, has been found to be associated with chronic pain.^22^ Furthermore, in the context of SPS, it is generally recommended to assess factors of the family and social environment. Highly sensitive adolescents tend to benefit disproportionately from positive and supportive contexts, however, exposure to negative social environments can have a heightened impact on them.^23–25^ In mid-adolescence, the social environment is primarily shaped by contact with peers and the school, while parents and relationship to parents still play an important role.^26^ Research shows the influence of parenting which can shape the well-being and development of highly sensitive children and adolescents.^27^,^28^ Thus, understanding and addressing both the peer and family contexts is essential.

Quantitative sensory testing (QST; pain elicited experimentally, eg, through heat or cold) is one way to test for altered sensory functioning in response to painful stimuli, as it provides information about pain tolerance and pain threshold.^29^ QST consists of static tests, which are used to determine pain thresholds, as well as dynamic tests investigating central processing of painful sensations (eg, conditioned pain modulation).^30^ Usually, pain is rated with regard to intensity and unpleasantness, two important dimensions of pain responsivity that are influenced by various factors, such as age and coping.^31^ QST is a method used to estimate somatosensory functionality.^32^ Altered pain modulation, as determined through QST, has been identified in pediatric samples with, for example, migraine,^33^ chronic back pain (in patients with adolescent idiopathic scoliosis),^34^ and complex regional pain syndrome.^35^

The aim of the present study was to evaluate individual differences in SPS and their influence on pain perception in a healthy sample of adolescents. We focused on mid-adolescence (16–19 years),^36^ an age range often overlooked in research that inquires factors that impact pain and pain-related outcomes.^37^ Our goal was to experimentally test (1) differences between sensitivity groups with regard to pain tolerance and pain threshold; (2) whether induced emotional state (positive, negative, neutral) would influence subsequent measured (ie, in °C) and self-reported (ie, ratings on a scale from 0 to 10) ratings for pain tolerance and pain threshold, and (3) if emotional regulation, parental bonding, and social support serve as potential moderators in relation to the link between sensitivity and pain perception.

## Methods

### Study Design

Between June 2021 and April 2022, an experimental study with healthy adolescents was conducted at the Faculty of Psychology, University of Basel, Switzerland. Statistical analysis estimated that a sample size of N = 128, with a power of 0.8, an alpha-error of 5%, and a beta-error of 20%, would be necessary to detect a medium effect size of f = 0.25 between groups. Written informed consent was obtained from each participant before their participation in the study. Since the risk of participation in this study can be described as low, Swiss law allows adolescents aged 14 and over who are capable of judgment to sign the consent form without parental consent.^38^ The Local Ethics Committee of the Canton Basel, Switzerland, approved the study (EKNZ Project ID: 2020–03027). The study is in compliance with the Declaration of Helsinki^39^ and registered at Clinical.Trials.gov: NCT04473014.

### Study Population

Healthy participants between the ages of 16 and 19 years with good knowledge of German were recruited. Participants were excluded if they had a chronic condition (including chronic pain, as participants with chronic pain may have altered sensory processing), skin pathologies, or sensory abnormalities affecting the tactile or thermal modality; if they were currently taking any kind of medication that could affect their pain perception at the moment of testing, or if they were in psychological or psychiatric treatment. Participants were compensated with cash and reimbursement of their travel costs.

Study participants were recruited through a combination of outreach efforts, including at local public high schools and advertising on the University’s website. In accordance with the ethical guidelines, the topic of high sensitivity was mentioned in the advertisement. Interested participants could access the study through a QR code provided on the advertisement which brought them directly to an online platform to schedule their individual appointment. Following this, a research assistant emailed them to confirm the appointment and sent them a document with comprehensive study-related information. This information included the location and duration of the appointment, exact experimental procedure, as well as details concerning the heat pain device and questionnaires that participants would complete. The document also outlined potential risks associated with participation, aim of the study, reimbursement, data protection and privacy policies, and emphasized the voluntary nature of participation. All documents were provided in German.

### Study Procedure

The experiment was carried out by research assistants at a Master’s level in psychology. Upon arrival in the lab, participants had the opportunity to ask questions, then signed the consent forms, and completed the questionnaires (described further under *Measures and Questionnaires*) on a tablet, followed by a trial heat pain exposure, as well as baseline pain threshold and tolerance assessment. After that, they were asked to watch a series of 20 pictures from the International Affective Picture System (IAPS).^40^ Participants were randomly allocated to view positively (N = 34), negatively (N = 33), or neutrally (N = 33) valenced pictures, using the built-in random number generator in Microsoft Excel. Positively valenced pictures show pleasant sceneries, such as three puppies looking at the camera, an example of neutral pictures would be a person reading on a chair, and negatively valenced pictures were scary or brutal, such as a plane crash scene. The study investigator was aware of the allocation and selected the corresponding IAPS set accordingly.

After each picture, participants were asked to rate the picture on three emotional dimensions: pleasure (ie, from happy to unhappy), arousal (ie, from excited to relaxed), and dominance (ie, from in control to dominated). The rating was done with the Self-Assessment Manikin (SAM), an affective rating system where a graphic figure depicts values along each of the three dimensions on a continuously varying scale.^41^ A test trial with one picture was done before the experiment started to ensure that participants understood the different dimensions and the rating system.

Following the IAPS pictures, with a duration of under five minutes, the second pain threshold and pain tolerance assessment took place. After that, participants were debriefed about the aims of the study, and received their reimbursement.

### Heat Pain Threshold and Tolerance

The heat stimuli were administered to the right volar forearm using the Thermo Sensory Analyser (TSA-II; Medoc, Israel), a commonly used and safe device. The thermode was positioned at different locations for the pre- and post-measurements to avoid potential effects of sensitization or habituation.^42^ Standardized testing instructions were used across participants. Pain threshold was determined with the self-controlled search method starting at a baseline temperature of 32°C, with a mouse-click inducing a rise of 0.5°C per click. Participants were asked to increase the magnitude of the heat stimulus by themselves to the point where they felt it changing from “hot” to “painful”.^43^ To prevent injuries, the measurement automatically stopped at a temperature of 52°C. Once the individual pain threshold has been reached, the device quickly resumed to its baseline temperature. Pain threshold was measured three times before the emotion induction (ie, baseline measurement), and three times after the emotion induction (ie, post measurement), and defined as the average of the three measurements (baseline and post). Pain tolerance was defined as the time in seconds elapsed from onset of the heat stimulus at 32°C to the participant’s withdrawal from the stimulus and was determined by the methods of limits. Temperature increased by 0.5°C per click and participants were instructed to stop the increasing heat stimulus once it became too painful to stand any longer.^44–46^ Pain tolerance was measured three times at baseline and three times at post measurement. To minimize interference between pain tolerance and threshold, pain threshold was assessed before pain tolerance.^43^ A test run was done before the start of the baseline measurement to familiarize participants with the device. Participants were asked to rate pain intensity and unpleasantness after each pain threshold and pain tolerance stimulation on a visual analogue scale (VAS), ranging from 0 (ie, not at all painful/unpleasant) to 10 (ie, worst pain imaginable/most unpleasant imaginable). Throughout the manuscript, we will refer to the results in °C as “measured”, and the VAS rating as “self-reported” outcomes, to differentiate between the two.

### Measures and Questionnaires

Demographic information, including date of birth, gender, current living situation, current school, and language(s) spoken at home were assessed. German translations were used for all questionnaires.

The calculation of all Cronbach alphas is based on the questionnaires completed before the start of the experimental procedure.

#### Sensitivity

Sensitivity was assessed by means of the Highly Sensitive Child Scale (HSC), a self-report questionnaire with 12 items that has been validated in children and adolescents aged 8–19 years in English. The German version has not yet been validated.^15^ Participants rate each item on a 7-point Likert-scale ranging from 1 (=not at all) to 7 (=extremely). In addition to a total score, three subscales can be calculated: Ease of Excitation (EOE, 5 items, eg, “I get nervous when I have to do a lot in little time”), Aesthetic Sensitivity (AES, 4 items, eg, “I love nice smells”), and Low Sensory Threshold (LST, 3 items, eg, “I don’t like watching TV programs that have a lot of violence in them”). Higher scores indicate higher sensitivity. Participants were divided into three different groups (low, medium, and high sensitivity) based on the upper and lower 30%, and the 40% in the middle.^15^

Cronbach’s alpha calculated on the basis of the baseline questionnaire was 0.67 for the EOE subscale (5 items), 0.49 for the AES subscale (4 items), 0.48 for the LST subscale (3 items), 0.69 for the HSC total score in the current sample and were comparable to those reported in the original validation study.^15^

#### Emotion Regulation

The Emotion Regulation Questionnaire (ERQ)^47^ is a validated^48^ 10-item self-report questionnaire designed to assess the habitual use of two emotion regulation strategies, namely Cognitive Reappraisal (eg, “When I’m faced with a stressful situation, I make myself think about it in a way that helps me stay calm”) and Expressive Suppression (eg, “I keep my emotions to myself”). Items are rated on a 7-point Likert-scale ranging from 1 (=strongly disagree) to 7 (=strongly agree). Higher scores indicate more habitual use of the respective emotion regulation strategy.

Cronbach**’**s α was 0.69 for the cognitive reappraisal subscale (6 items) and 0.71 for the expressive suppression subscale (4 items) in this sample, similar to the values in our previous study.^17^

#### Parenting Style

The Parental Bonding Instrument (PBI)^49^ is a validated^50^ 12-item questionnaire that examines the child’s subjectively remembered parenting styles retrospectively on the two dimensions *care* and *protection*. Items are rated for both parents individually and scored on a 4-point Likert-scale ranging from “3 (=very like) to 0 (=very unlike)”.

Cronbach’s α was 0.85 for the care subscale (12 items) for the mother and 0.89 for the father. For the protection subscale (13 items), Cronbach’s α was 0.84 for both mothers and fathers.

#### Social Support

The Child and Adolescent Social Support Scale (CASSS)^51^ is a validated^52^ measure of perceived social support provided by parent, teacher, classmate, and best friend with 60 items, rated on two aspects: Frequency ratings use a 6-point Likert-scale ranging from 1 (=never) to 6 (=always), assessing how frequent support is being received from the respective party, while importance ratings use a 3-point Likert-scale ranging from 1 (=not important) to 3 (=very important) and assess how important said support is for the participant.

Cronbach’s α was 0.89 for the parent frequency subscale (12 items), 0.74 for the importance subscale (12 items); 0.89 for the teacher frequency subscale (12 items), 0.78 for the importance subscale (12 items); 0.87 for the classmate frequency subscale (12 items), 0.77 for the importance subscale (12 items); 0.90 for the close friend frequency subscale (12 items), 0.85 for the importance subscale (12 items).

## Statistical Analyses

All analyses were carried out using R Statistical Software (v4.1.2).^53^

For the classification of sensitivity groups, we used the “type 7” method with the quantile function from the stats package, as our data was skewed, which can significantly affect the accuracy and reliability of quantile calculations. The “type 7” method is well suited for dealing with skewed data because it considers both the lower and upper values in each quantile, making it robust to outliers and skewed ends.^54^

Descriptive statistics were used to summarize participant characteristics. To account for multiple testing and minimize the likelihood of false positives, we applied Bonferroni correction. Measured (heat pain tolerance and threshold in °C) and self-reported (intensity and unpleasantness ratings) pain measures were averaged across the three measurement time points prior to mood induction to establish an overall baseline variable. The same was done with the three post measurement points. Violin plots were used to describe the distribution of the HSC subscales (EOE, AES, and LST) and the mean values of the pain indicators reported at baseline (tolerance, threshold, and VAS scales for unpleasantness and intensity). Spearman correlations were calculated, due to variables not being normally distributed, to examine associations between the three subscales of the HSC scale, emotion regulation, parental bond, social support, and pain variables.

To determine the effects of the experiment, regression models were applied to calculate the effect of the interaction between the mood induction group (neutral, negative, positive) and HSC-based sensitivity group (low, medium, high sensitivity) on the measured and self-reported heat pain outcomes. The neutral mood induction group was used as the reference group. Age, gender, pain threshold and tolerance scores at baseline were included as control variables.

Multiple regression models were computed to examine the relationship between measured and self-reported heat pain variables and the three HSC subscales, controlling for age and gender. Subsequently, potentially moderating factors (emotion regulation, parental attachment, and social support) were included as interaction effects.^55^

Multiple 1-way analyses of variance (ANOVAs) were then calculated to determine differences between the three sensitivity groups regarding emotion regulation, parental attachment, social support, measured and self-reported pain variables. Where ANOVAs indicated statistically significant differences, post hoc Tukey’s HSD tests were performed to identify specific group differences.

## Results

### Sample Characteristics

A total of 100 healthy adolescents consented to participate, completed the experiment, and were included in the analyses. Of those, 71% identified as female and 29% as male, M_age_ 17.62 years (SD = 3.15). Most participants (93%) were going to school (public high school, university, and applied university) and lived with both of their parents (69%; see Table 1). Significant group differences were observed in the VAS threshold intensity variable among the randomly assigned mood induction groups, prior to the experiment. Correlations between included variables and violin plots of the sensitivity and pain variables can be found in the Supplement (stable 1 and sfigure 1). Descriptive statistics on the sensitivity and pain variables, disaggregated by the three mood induction groups can be found in the Supplement (stable 2).

**Table 1:** Sample Characteristics

Variable	*Total Sample* (N *= 100)*
Gender female, n (%)	71 (71)
Age, *M* (*SD*)	17.62 (3.15)
Attending high school or (applied) university, n (%)	93 (93)
Apprenticeship, n (%)	4 (4)
Unemployed, n (%)	3 (3)
Housing situation	
Living with both parents, n (%)	69 (69)
Living with one parent, n (%)	23 (23)
Living in two households, n (%)	5 (5)
Living alone, n (%)	3 (2)
Sensory Sensitivity (HSC)	
Total score, *M* (*SD*)	4.62 (0.68)
Ease of excitation, *M* (*SD*)	4.29 (1.05)
Aesthetic sensitivity, *M* (*SD*)	5.77 (0.75)
Low sensory threshold, *M* (*SD*)	3.61 (1.16)
Low sensitivity group (30% quantile), n (%)	32 (32)
Medium sensitivity group (40% quantile), n (%)	36 (36)
High sensitivity group (30% quantile), n (%)	32 (32)
Baseline	
VAS threshold intensity, *M* (*SD*)	24.84 (103.23)
VAS threshold unpleasantness, *M* (*SD*)	4.02 (2.72)
VAS tolerance intensity, *M* (*SD*)	13.58 (46.79)
VAS tolerance unpleasantness, *M* (*SD*)	6.27 (2.67)
Heat pain tolerance, *M* (*SD*)	48.74 (1.53)
Heat pain threshold, *M* (*SD*)	45.84 (2.79)
Emotion regulation (ERQ)	
Cognitive reappraisal, *M* (*SD*)	4.55 (0.99)
Expressive suppression, *M* (*SD*)	3.79 (1.26)
Child and adolescent social support scale (CASSS)	
Parents importance, *M* (*SD*)	1.12 (0.31)
Parents frequency, *M* (*SD*)	3.2 (0.87)
Teacher importance, *M* (*SD*)	1.17 (0.38)
Teacher frequency, *M* (*SD*)	3.14 (0.92)
Classmates importance, *M* (*SD*)	1.07 0.43
Classmates frequency, *M* (*SD*)	3.44 (0.87)
Best friend importance, *M* (*SD*)	1.42 (0.38)
Best friend frequency, *M* (*SD*)	1.42 (0.38)
Parental bonding instrument (PBI)	
Mother overprotection, *M* (*SD*)	10.68 (6.49)
Father overprotection, *M* (*SD*)	9.25 (6.41)
Mother care, *M* (*SD*)	27.76 (6.13)
Father care, *M* (*SD*)	25.08 (7.38)

**Notes**: M and SD are used to represent mean and standard deviation.

**Abbreviations**: HSC, Highly Sensitive Child Scale; ERQ, Emotion regulation; CASSS, Child and Adolescent Social Support Scale; PBI, Parental Bonding Instrument.

### Differences Between Sensitivity Groups Regarding Measured Pain Tolerance and Threshold

**Figure 1:**
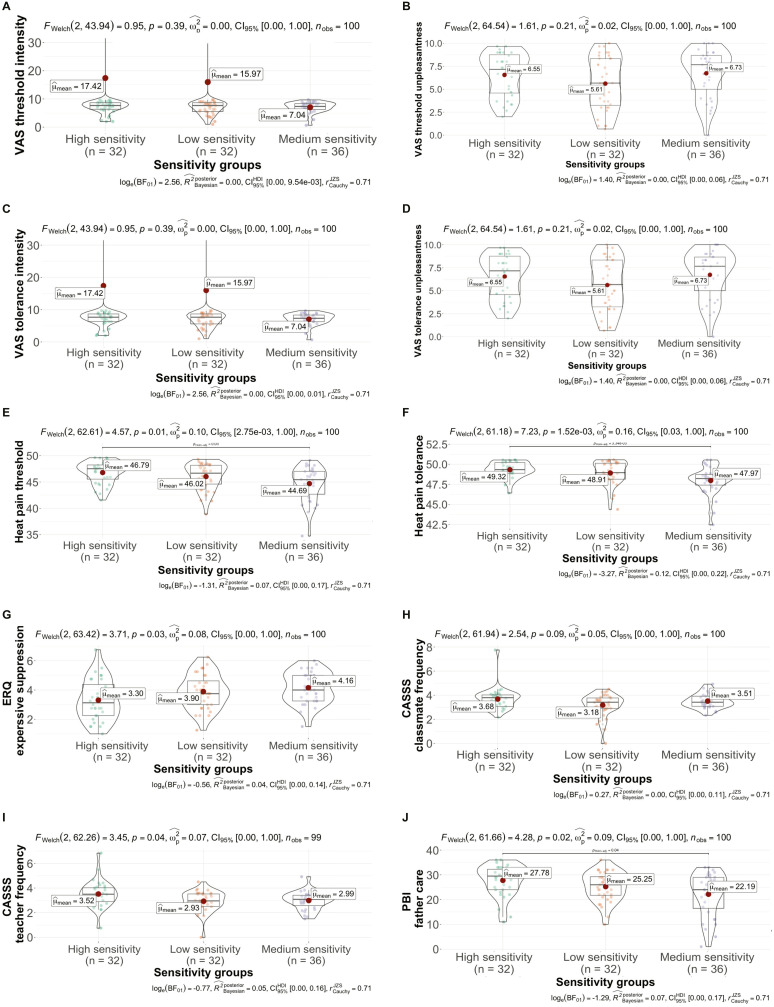
Visualization of mean differences between sensitivity groups.Violin plots displaying the distribution of scores across sensitivity groups (low, medium, and high) for baseline pain threshold, pain tolerance, and scores on the ERQ, CASSS, and PBI. Each violin plot visualizes the density and spread of data within groups, with overlaid mean values and 95% confidence intervals. Statistical significance was assessed using Welch’s ANOVA, and post-hoc comparisons indicate significant pairwise differences between groups, with adjusted p-values and effect sizes shown for each significant comparison.

**Notes**: (**A**) Comparison between sensitivity groups regarding VAS threshold intensity. (**B**) Comparison between sensitivity groups regarding VAS threshold unpleasantness. (**C**) Comparison between sensitivity groups regarding VAS tolerance intensity. (**D**) Comparison between sensitivity groups regarding VAS tolerance unpleasantness. (**E**) Comparison between sensitivity groups regarding heat pain threshold. (**F**) Comparison between sensitivity groups regarding heat pain tolerance. (**G**) Comparison between sensitivity groups regarding expressive suppression. (**H**) Comparison between sensitivity groups regarding classmate frequency. (**I**) Comparison between sensitivity groups regarding teacher frequency. (**J**) Comparison between sensitivity groups regarding father care.

**Abbreviations**: ERQ, Regulation Questionnaire; CASSS, Child and Adolescent Social Support Scale; PBI, Parental Bonding Instrument.

Differences between the three sensitivity groups were found for the measured heat pain variable pain threshold at baseline (F_(2,97)_ = 5.009, *p* < 0.01), with the low-sensitivity group scoring the highest 46.79 (*SD* = 2.15)), followed by the medium sensitivity group (*M* = 46.02, *SD* = 2.53) and the high-sensitivity group (*M* = 44.69, *SD* = 3.27). Post-hoc comparisons using Tukey’s HSD test revealed that the high-sensitivity group had a significantly lower baseline pain threshold compared to the lower group, with a mean difference of −2.10 (95% CI [−3.69, −0.50]), *p* = 0.007. However, no significant difference was found between the medium group and the lower group (mean difference = −0.77, 95% CI [−2.32, 0.79], *p* = 0.471) or between higher and the medium group (mean difference = −1.33, 95% CI [−2.88, 0.22], *p* = 0.108). This was also true for baseline heat pain tolerance (F_(2,97)_ = 7.431, *p* < 0.001), with the low-sensitivity group scoring the highest (*M* = 49.32, *SD* = 0.98), followed by the medium group (*M* = 48.91, *SD* = 1.48, and finally the high-sensitivity group (*M* = 47.97, *SD* = 1.75; see Table 2 and Figure 1). More information on differences between sensitivity groups can be found in the Supplement. Post-hoc comparisons using Tukey’s HSD test revealed that the higher sensitivity group had a significantly lower baseline pain tolerance compared to the lower group, with a mean difference of −1.35 (95% CI [−2.20, −0.49]), *p* < 0.001. The higher group also had a significantly lower baseline pain tolerance compared to medium group, with a mean difference of −0.94 (95% CI [−1.77, −0.11]), *p* = 0.022. However, there was no significant difference between the medium and lower (mean difference = −0.41, 95% CI [−1.24, 0.42], *p* = 0.477).

**Table 2:** Fixed-Effects ANOVA Concerning Sensitivity Group Differences

Predictor	Sum of Squares	*df*	Mean Square	*F*	*p*	_partial_ η^2^	_partial_ η^2^ 90% CI [LL, UL]
Baseline Heat pain tolerance							
(Intercept)	236755.01	1	236,755.01	114,524.15	0.000		
Sensitivity group	30.72	2	15.36	7.43	0.001	0.13	[0.04, 0.23]
Error	200.53	97	2.07				
Baseline Heat pain threshold							
(Intercept)	209453.54	1	209,453.54	29,068.35	0.000		
Sensitivity group	72.19	2	36.09	5.01	0.009	0.09	[0.01, 0.18]
Error	698.94	97	7.21				

**Notes**: LL and UL represent the lower-limit and upper-limit of the partial η^2^ confidence interval, respectively. *p* indicates p-value.

### Influence of Emotional State on Subsequent Pain Ratings

No significant differences regarding pain ratings were found between sensitivity groups based on mood induction. The interaction between the three experimental groups and the HSC total score did not differ in terms of measured heat pain tolerance and threshold at post assessment. Results differed in terms of self-reported VAS ratings for pain tolerance and unpleasantness after the experimental procedure, with a positive interaction coefficient of the positive mood induction group, indicating that participants in the positive mood induction group with higher HSC total scores reported higher pain ratings on the VAS tolerance-unpleasantness scale (*b* = 1.140, *p* < 0.05). The same results were obtained for the HSC subscales EOE (*b* = 0.734, *p* < 0.05) and LST (*b* = 0.680, *p* < 0.05), whereas the AES subscale did not reach a significant level (see Table 3). Interaction plots of sensitivity and pain variables combined with emotion regulation, social support, and parental bonding can be found in the Supplement (sfigure 2).

**Table 3:** Regression Results Using Post Mood Induction VAS Heat Pain Tolerance and Unpleasantness as the Criterion

Predictor	*b*	*b* 95% CI [LL, UL]	*sr^2^*	*sr^2^* 95% CI [LL, UL]	Fit
**Model 1:** HSC total score					
(Intercept)	4.47**	[1.99, 6.95]			
Negative mood induction group	−2.51	[−6.17, 1.15]	0.00	[−.00, 0.01]	
Positive mood induction group	−4.79**	[−8.20, −1.37]	0.01	[−.00, 0.03]	
Total score (HSC)	−0.95**	[−1.43, −0.47]	0.02	[−.00, 0.04]	
Age	−0.01	[−0.08, 0.06]	0.00	[−.00, 0.00]	
Gender, female	−0.15	[−0.63, 0.33]	0.00	[−.00, 0.00]	
Baseline heat pain VAS tolerance unpleasantness	0.94**	[0.86, 1.02]	0.81	[0.72, 0.91]	
Negative mood induction group*total score (HSC)	0.67	[−0.13, 1.46]	0.00	[−.01, 0.01]	
Positive mood induction*total score (HSC)	1.14*	[0.42, 1.86]	0.01	[−.00, 0.03]	
					*R^2^* = 0.870**
					95% CI[0.81, 0.89]
**Model 2:** EOE subscale					
(Intercept)	2.25*	[0.46, 4.03]			
Negative mood induction group	−1.30	[−3.72, 1.13]	0.00	[−.00, 0.01]	
Positive mood induction group	−2.69*	[−4.79, −0.59]	0.01	[−.00, 0.02]	
Ease of excitation (HSC)	−0.52**	[−0.82, −0.22]	0.02	[−.00, 0.04]	
Age	−0.00	[−0.07, 0.07]	0.00	[−.00, 0.00]	
Gender, female	−0.20	[−0.69, 0.28]	0.00	[−.00, 0.01]	
Baseline heat pain VAS tolerance unpleasantness	0.94**	[0.86, 1.02]	0.81	[0.72, 0.90]	
Negative mood induction group*ease of excitation (HSC)	0.43	[−0.12, 0.97]	0.00	[−.01, 0.01]	
Positive mood induction*ease of excitation (HSC)	0.73*	[0.27, 1.20]	0.01	[−.00, 0.03]	
					*R^2^* = 0.866**
					95% CI[0.80, 0.89]
**Model 3:** LST subscale					
(Intercept)	2.14*	[0.38, 3.90]			
Negative mood induction group	−0.80	[−2.56, 0.95]	0.00	[−.00, 0.01]	
Positive mood induction group	−1.96*	[−3.72, −0.19]	0.01	[−.01, 0.02]	
Low sensory threshold (HSC)	−0.57**	[−0.91, −0.22]	0.02	[−.00, 0.04]	
Age	−0.02	[−0.09, 0.05]	0.00	[−.00, 0.00]	
Gender, female	−0.09	[−0.58, 0.41]	0.00	[−.00, 0.00]	
Baseline heat pain VAS tolerance unpleasantness	0.95**	[0.87, 1.03]	0.80	[0.71, 0.90]	
Negative mood induction group*low sensory threshold (HSC)	0.39	[−0.10, 0.88]	0.00	[−.01, 0.01]	
Positive mood induction*low sensory threshold (HSC)	0.68*	[0.23, 1.13]	0.01	[−.00, 0.03]	
					*R^2^* = 0.864**
					95% CI[0.80, 0.89]

**Notes**: A significant *b*-weight indicates the semi-partial correlation is also significant. *b* represents unstandardized regression weights. *sr^2^* represents the semi-partial correlation squared. *LL* and *UL* indicate the lower and upper limits of a confidence interval, respectively. *indicates p < 0.05. **indicates p < 0.01.

**Abbreviations**: HSC, Highly Sensitive Child Scale; EOE, Ease of Excitation (HSC subscale); LST, Low Sensory Threshold (HSC subscale).

### Prediction of Self-Reported and Measured Heat Pain Variables

None of the self-reported heat pain indicator variables reached significance. With respect to measured heat pain (as measured in °C), EOE was a significant predictor of lower heat pain threshold (*b* = −0.830, *p* < 0.01) and tolerance (*b* = −0.428, *p* < 0.05). This was also the case for the LST subscale, which significantly predicted a lower heat pain threshold (*b* = −0.630, *p* < 0.05), but not tolerance, following Bonferroni correction (*b* = −0.404, *p* < 0.05). In contrast, the AES subscale was not a significant predictor of either measured or self-reported heat pain variables (see Table 4).

**Table 4:** Regression Results Using Baseline Heat Pain Threshold and Tolerance (in °C) as the Criterion

Baseline heat pain threshold					
Predictor	*b*	*b* 95% CI [LL, UL]	*sr^2^*	*sr^2^* 95% CI [LL, UL]	Fit
(Intercept)	52.22**	[48.36, 56.09]			
Ease of excitation (HSC)	−0.83**	[−1.33, −0.33]	0.10	[−.01, 0.21]	
Age	−0.13	[−0.30, 0.04]	0.02	[−.03, 0.08]	
Gender, female	−0.66	[−1.82, 0.51]	0.01	[−.03, 0.05]	
					*R^2^* = 0.130**
					95% CI[0.02, 0.24]
(Intercept)	50.77**	[47.14, 54.41]			
Low sensory threshold (HSC)	−0.63*	[−1.11, −0.15]	0.06	[−.03, 0.16]	
Age	−0.13	[−0.31, 0.04]	0.02	[−.03, 0.08]	
Gender, female	−0.39	[−1.61, 0.82]	0.00	[−.02, 0.03]	
					*R^2^* = 0.098*
					95% CI[0.00, 0.20]
Baseline heat pain tolerance					
(Intercept)	51.49**	[49.35, 53.63]			
Ease of excitation (HSC)	−0.43*	[−0.71, −0.15]	0.09	[−.02, 0.19]	
Age	−0.03	[−0.13, 0.06]	0.00	[−.02, 0.03]	
Gender, female	−0.47	[−1.11, 0.18]	0.02	[−.03, 0.07]	
					*R^2^* = 0.112**
					95% CI[0.01, 0.22]
(Intercept)	51.00**	[49.03, 52.97]			
Low sensory threshold (HSC)	−0.40*	[−0.66, −0.15]	0.09	[−.02, 0.19]	
Age	−0.03	[−0.13, 0.06]	0.00	[−.02, 0.03]	
Gender, female	−0.29	[−0.95, 0.37]	0.01	[−.02, 0.04]	
					*R^2^* = 0.114**
					95% CI[0.01, 0.22]

**Notes**: A significant *b*-weight indicates the semi-partial correlation is also significant. *b* represents unstandardized regression weights. *sr^2^* represents the semi-partial correlation squared. *LL* and *UL* indicate the lower and upper limits of a confidence interval, respectively. * indicates p < 0.05. ** indicates p < 0.01.

**Abbreviation**: HSC, Highly Sensitive Child Scale.

### The Moderating Influence of Emotion Regulation, Social Support, and Parental Bonding

Overall, no consistent significant predictors of measured and self-reported pain were found. Also, no significant interactions were found between pain indicators (measured and self-reported), cognitive reappraisal (ERQ), parenting importance (CASSS), parenting frequency (CASSS), and maternal care (PBI). The emotion regulation subscale expressive suppression showed a significant and negative interaction with the HSC subscale LST. Regarding social support, the CASSS subscale best friend showed a negative interaction effect with the HSC subscale AES on several pain variables. Fatherly overprotection positively interacted with the HSC subscales AES and LST regarding VAS tolerance and intensity and heat pain tolerance. A table with Spearman correlations between all variables and more information on moderating variables can be found in the Supplement (stable 1).

## Discussion

We set out to investigate the effect of SPS on pain perception in a sample of healthy adolescents. Experimental pain can be used to examine the response to painful stimuli and has critically advanced the understanding of the mechanisms, assessment and treatment of pain in healthy and clinical samples.^56^ Pain induction contributes to an understanding of measured pain tolerance and threshold as well as self-reported ratings of associated intensity and unpleasantness. These values provide information about differences in sensory functioning such as altered pain modulation.^29^,^33–35^ Consequently, a study has shown that patients experiencing chronic pain had lower pain threshold and tolerance compared to a healthy control group.^57^ In this study, pain sensitivity was defined as reduced pain threshold and tolerance across different experimental pain stimulus modalities. Our own analysis yielded a high number of non-significant results, yet it also provided valuable insights into the relationship between SPS and pain perception in adolescents. While certain hypothesized effects did not emerge, our results nevertheless contribute to a more nuanced understanding of the subject.

In our experiment, we examined whether induced emotional states have an impact on subsequent pain tolerance and pain threshold. Contrary to our hypotheses, there was no significant effect of mood induction on participants’ pleasure, arousal, or dominance ratings across the three mood conditions. Several potential factors could have contributed to this outcome. First, research suggests that the association between visual stimuli (eg, pictures) and emotional ratings is stronger in older than in young adults.^58^ Therefore, as an experimental mood induction, IAPS may not be as affective in adolescents as compared to older individuals. In addition, cohort-based shifts in perceptions of IAPS imagery may have minimized effects, as 21^st^ century youth has become more attuned to publicly available, negatively valenced images such as of violence and self-injury.^59^ This exposure may lead to desensitization regarding affective reactions and physical arousal.^60^ Further, some researchers have expressed doubts about the neutral IAPS pictures, which may not be neutral after all, but rather evoke ambivalent emotional states.^61^ Ambivalence, among other things, can ignite feelings of uncertainty,^62^ which has been found to be associated with increased pain perception.^63^

Another possible explanation for our finding might be that the heat sensations have not yet been ideally applied with regard to duration, frequency, and interval.^56^ Unlike for other types of pain stimuli, there are few established guidelines for heat pain stimuli.^56^ The lack of standardized protocols and recommendations for these parameters in heat pain studies might lead to significant variability across studies and hence compromise the effectiveness of the pain induction method.

Additionally, we wanted to examine differences between low, medium and high-sensitivity groups in relation to pain tolerance and threshold. Interestingly, our findings revealed noteworthy differences between measured and self-reported pain outcomes: The HSC score was predictive of the *measured* pain outcomes (ie, pain tolerance and threshold in °C), while no significant and consistent correlations between high sensitivity and *self-reported* pain ratings were found. Participants with higher sensitivity scores demonstrated lower pain threshold and pain tolerance compared to the other groups. Thus, no linear relationship between measured and self-reported pain perception could be found in our data, as for the latter, the low, medium, and high-sensitivity group did not differ consistently. In other words, adolescents with higher sensitivity rate the highest tolerable heat stimulus as equally intense and unpleasant as those with lower sensitivity. However, the pain threshold of more sensitive adolescents is at a lower point on the temperature scale. It therefore appears that the physical sensation and possibly the activation of peripheral nociceptive receptors is influenced by sensitivity, but the rating is not. This might be due to the distinction between sensitivity and responsivity: While sensitivity describes aspects of perception and internal processing of external influences (ie, the input), responsivity refers to the resulting behavioral consequences (ie, the output).^11^ The differences in responsivity are indeed conditioned by differences in environmental sensitivity, but the two constructs cannot be considered the same.^64^ Translated to our experimental setting, varying threshold, and tolerance across groups regarding the induced *measured* heat stimulus could be explained by divergent sensitivity of participants, which significantly varied between the groups. The *self-reported* pain ratings, on the other hand, would be based on inter-individual differences in reactivity that remained consistent despite different pain characteristics.

Lastly, previous research suggests that individuals with higher HSC scores are more affected by environmental factors than those with lower sensitivity.^15^ This is also illustrated in the botanical metaphor, where orchids, tulips, and dandelions represent the three sensitivity groups and their relationship to environmental circumstances.^16^ In our study, we included measures such as emotional regulation, parental bonding, and social support to assess environmental influences.

Although some significant interactions between these variables were found, they were not consistent, suggesting that while SPS may interact with social and emotion-regulatory factors, the specific nature and direction of these relationships remain complex. Additionally, ratings on the parental bonding assessment scale *care* were slightly above the average compared to those reported in previous studies.^49^,^65^ Future studies could further examine how these environmental factors interact with sensitivity using a combination of methods beyond self-report.^66^,^67^ Additionally, a recent study demonstrated the potential impact of parental socioeconomic factors on adolescents’ pain, which could have been valuable to consider in our assessments.^68^

## Strengths and Limitations

This study has several strengths. To the best of our knowledge, this is the first study to explore SPS in the context of experimental pain in adolescents. Also, validated assessments for mood induction as well as a standardized heat pain application were chosen. Further, we included measures of emotional and social factors, which allowed us to test for various contextual influences.

Despite these strengths, our study also comes with several limitations: Mentioning sensitivity in the advertisement may have introduced a certain bias by encouraging self-selection among participants. Next, measuring pain using VAS ratings has been the subject of several concerns in past research.^69^,^70^ Hence, undesirable effects of the scale cannot be ruled out in this study. According to our power analysis, we determined a moderate level of statistical power but unfortunalely,^71^ our desired sample size could not be achieved in part due to COVID-19 pandemic constraints. This indicates a reasonable probability of detecting the hypothesized effects within our regression analysis. Nevertheless, increasing sample size in future studies offers opportunities to increase the sensitivity of detecting statistically significant effects. Further, Cronbach’s alphas for the HSC subscales AES and LST were low, whereas the alphas for the total score, the EOE subscale and the ERQ were acceptable.^71^ Generally, it is important to note that, given the influenceability of this measure, Cronbach’s alpha can only be considered as one indicator of the internal consistency.^72^ More robust measures, such as McDonald’s omega, have already been proposed as alternatives to Cronbach’s alpha.^73^ The unsatisfactory values for the HSC subscales in our study may be attributed to the influence of the number of items across scales, and the small number of items per subscale in the calculation of alphas.^72^ Furthermore, the dimensionality of the scale may influence its alphas, as previous studies have raised concerns about the factor structure of the Highly Sensitive Person Scale (the adult version of the HSC), with some suggesting a single-factor solution and others suggesting a three-factor solution.^74^,^75^ Finally, the English scale was originally validated in the UK where slightly higher alpha values were reported.^15^ In this study, the German translation was used, which has yet to be validated. It is pertinent that future studies examine the construct validity of the HSC scale not only in healthy children and adolescents, but also in clinical samples. In addition, even before the experimental procedure began, there appeared to be significant group differences between the randomly assigned mood induction group, particularly in terms of the VAS threshold intensity variable.

## Conclusion and Future Directions

Our study aimed to experimentally investigate the role of sensory processing sensitivity in pain perception among adolescents focusing on differences in pain tolerance and threshold, and the effects of mood induction. A substantial difference in measured pain outcomes suggests higher pain vulnerability for more sensitive adolescents and supports the assumption that high sensitivity might be a potential risk factor for intensified pain perception. Assessing sensitivity in a clinical context could be particularly relevant, as highly sensitive individuals tend to benefit more from (preventative) interventions. Despite compelling theoretical links between high sensitivity and altered pain processing, the high number of non-significant results was surprising, particularly the lack of effect from mood induction and the absence of differences in self-reported pain ratings across sensitivity groups. Finally, the insights gained regarding sensitivity, emotion regulation, social support, and pain variables highlight the complexity of these relationships and suggest a need for further research to investigate underlying mechanisms.

## Data Availability

All study-related deidentified data will be shared upon request made to the corresponding author without time limit.
